# Perinatal mental health care in a rural African district, Uganda: a qualitative study of barriers, facilitators and needs

**DOI:** 10.1186/s12913-016-1547-7

**Published:** 2016-07-22

**Authors:** Juliet E. M. Nakku, Elialilia S. Okello, Dorothy Kizza, Simone Honikman, Joshua Ssebunnya, Sheila Ndyanabangi, Charlotte Hanlon, Fred Kigozi

**Affiliations:** Butabika Hospital, Makerere University, P.O.Box 24136 Kampala, Uganda; Makerere University, Kampala, Uganda; Department of Psychiatry and Mental Health, Perinatal Mental Health Project, Alan J Flisher Centre for Public Mental Health, University of Cape Town, Cape Town, South Africa; Ministry of Health, Kampala, Uganda; Department of Psychiatry, Addis Ababa University, College of Health Sciences, School of Medicine, Addis Ababa, Ethiopia; King’s College London, Institute of Psychiatry, Psychology and Neuroscience, Centre for Global Mental Health, London, UK

**Keywords:** Maternal mental health, Community mental health, Primary health care, Mental health services, Postnatal depression, Perinatal mental health

## Abstract

**Background:**

Perinatal mental illness is a common and important public health problem, especially in low and middle-income countries (LMICs). This study aims to explore the barriers and facilitators, as well as perceptions about the feasibility and acceptability of plans to deliver perinatal mental health care in primary care settings in a low income, rural district in Uganda.

**Methods:**

Six focus group discussions comprising separate groups of pregnant and postpartum women and village health teams as well as eight key informant interviews were conducted in the local language using a topic guide. Transcribed data were translated into English, analyzed, and coded. Key themes were identified using a thematic analysis approach.

**Results:**

Participants perceived that there was an important unmet need for perinatal mental health care in the district. There was evidence of significant gaps in knowledge about mental health problems as well as negative attitudes amongst mothers and health care providers towards sufferers. Poverty and inability to afford transport to services, poor partner support and stigma were thought to add to the difficulties of perinatal women accessing care. There was an awareness of the need for interventions to respond to this neglected public health problem and a willingness of both community- and facility-based health care providers to provide care for mothers with mental health problems if equipped to do so by adequate training.

**Conclusion:**

This study highlights the acceptability and relevance of perinatal mental health care in a rural, low-income country community. It also underscores some of the key barriers and potential facilitators to delivery of such care in primary care settings. The results of this study have implications for mental health service planning and development for perinatal populations in Uganda and will be useful in informing the development of integrated maternal mental health care in this rural district and in similar settings in other low and middle income countries.

## Background

Perinatal mental disorders are an important public health problem, especially in low- and middle-income countries (LMICs) [1]. These are mental disorders that occur either in pregnancy or the postpartum period and include antenatal and postnatal common mental disorders (CMD) and the severe mental disorders (SMD). Common mental disorders include major depression and anxiety disorders, such as generalized anxiety, panic and obsessive compulsive disorders, and phobias, such as social phobia. The severe mental disorders include bipolar disorder and the psychotic disorders including schizophrenia and delusional disorders.

Major depression, generalized anxiety, panic and obsessive compulsive disorders are more common during the first and third trimester of pregnancy. It is not uncommon for there to be co-occurrence of depression and anxiety symptoms. Bipolar and the psychotic disorders rarely have their onset in pregnancy but may occur as a result of relapse of a pre-existing disorder. During the postpartum period, mood disorders are by far the commonest mental disorders. These include the postpartum blues which are a mild and self-limiting form of postpartum distress, non-psychotic postpartum major depression and bipolar mania. Psychosis in the postpartum period is commonly manic in nature but may occur in form of psychotic depression, schizophrenia or an organic psychosis. These conditions frequently have their onset during the postpartum period although they may occur as a recurrence of an existing disorder. In this study we focus on depression and severe mental disorders in pregnancy and the postpartum period. The fifth edition of the Diagnostic and Statistical Manual of mental disorders [2] recognizes the occurrence of mood and psychotic disorders both in pregnancy and the postpartum period. It classifies these as disorders with peri-partum onset if the onset is during pregnancy or within four weeks of child birth. The manual defines major depression in the peri-partum period as a condition characterized by sad, empty or irritable mood accompanied by somatic and cognitive changes that significantly affect the individual’s capacity to function. Other symptoms include a fluctuating or labile mood and over-concern and intrusive thoughts about the infant’s wellbeing which may progress to frank delusions. The affected woman may also have severe anxiety that manifests in the form of fear of being alone with the infant. Postpartum onset psychotic disorders on the other hand are characterized by abnormal firmly held beliefs (delusions) centered on the baby, abnormal perception of the environment (hallucinations), disturbances in thinking, speech and behavior.

A recent systematic review indicated that the prevalence of perinatal mental disorders in low- and middle-income countries (LMICs) is higher than in high-income countries (HICs) [3]. Studies conducted in sub-Saharan Africa show variable prevalence estimates of common perinatal mental illness, particularly depression, ranging from 8.3 to 41 % in pregnancy [4–6], and from 3.5 to 34.7 % in the first year postpartum [7–9]. In Uganda, the prevalence of postpartum depression has been estimated to be 6.1 % among patients attending primary care clinics [10]. Although very little is known about the epidemiology of perinatal psychosis and other severe mental disorders in LMICs, the treatment gap for these disorders could be as high as with severe mental disorders at other times of the lifespan This high burden of mental ill health among perinatal women underscores the need for mental health services within maternal health care systems especially in sub-Saharan Africa.

Perinatal mental illness has been associated with poor maternal and neonatal outcomes [11]. Depression in pregnancy, for example has been shown to lead to adverse birth outcomes including preterm birth, lower birth weight [12] and reduced postpartum growth in the newborn. Post-partum depression on the other hand may lead to poor cognitive outcomes and psychiatric morbidity in childhood and adolescence [13–16]. In addition, depression during the perinatal period can cause impairment in work and family settings for the mother and can impact on a woman‘s ability to care for, nurture and respond to her infant [17]. Promotion of perinatal mental health, therefore, has the potential to reduce the risk of such morbidity among children and adolescents in the future.

There are very few studies regarding mental health care integrated into reproductive health care in low- and lower-middle income countries of sub-Saharan Africa. Studies in these countries highlight the barriers to mental health service delivery in general which may also be relevant for mental health service delivery to perinatal women. Such barriers include negative community attitudes that stigmatize women with mental ill health, inadequate infrastructure such as health facilities that are far and difficult to reach, social and economic disadvantage among women, including poverty, and personal or psychological barriers [18–20]. Less is known about the specific barriers to mental health care for perinatal women. One potential barrier is the excessive focus on the physical health of mothers, as is the case in many primary care settings, which leads to poor recognition of mental ill health among perinatal women by primary health care workers, causing a significant gap between need and service delivery [20]. The World Health Organization (WHO) recommends that in order to increase access to perinatal mental health care, this should be integrated into primary health care [21]. However, a vertical approach to service design, as is the case in Uganda, where maternity services and mental health services are traditionally provided separately, makes integration of services and access a challenge.

Exploration of barriers, facilitators and needs for perinatal mental health services is therefore important for informing programs intended to improve perinatal mental health services in a resource limited setting. This study aimed to explore and understand the factors that affect access to mental health care among pregnant and postpartum women in a rural, low resourced district of Uganda. Specifically, the objective was to explore the available knowledge and knowledge gaps, barriers and facilitators as well as perceptions of feasibility and acceptability of delivering mental health care to pregnant and postpartum women suffering from perinatal depression and psychosis in primary care settings. This study comes ahead of planning an intervention to address the large treatment gap, which has been estimated generally for mental health problems, to be over 80 % in low and middle income countries (LMICs) [22]. The study was carried out as part of formative work for the Program for Improving Mental health care (PRIME) [23]. PRIME is a research consortium evaluating the integration of mental health into primary and maternal health care in five low and middle income countries including Uganda, Ethiopia, Nepal, India and South Africa.

## Methods

### Study design

A qualitative study was conducted using focus group discussions and key informant interviews. This was necessitated by the paucity of qualitative enquiry into care, user and providers' attitudes, experiences and suggestions pertaining to perinatal mental health care in Uganda. In particular, for the Ugandan setting, it was considered critical to understand the rich perspectives of these key stakeholders in order to inform the future development of responsive and relevant services for the PRIME study.

### Setting

The study was carried out in Kamuli district in Eastern Uganda, 140 kilometers from the capital city, Kampala. The district is a densely populated, multi-cultural farming community with a population of nearly 500,000 people. Most people are ethnically Basoga although there are other tribes such as the Bagwere, Baganda and Balamogi. The majority of the population is engaged in subsistence farming. The literacy rate is lower than the national average of 78 % [24]. The health system consists of two district hospitals (one public and another private) and 64 primary health care centers (PHCs) with varying levels of care. The health facilities are graded as health center (HC) I, II, III and IV. The district hospitals serve as the referral points for the lower health centers. The HC IV (Health sub district) and HCIII all have a maternity unit in a building separate from the general health clinics, but within the same compound. The maternity units are staffed with midwives who are the main health care providers for perinatal women. All perinatal women are triaged through the outpatient departments of health facilities to the maternity units. Both antenatal and postnatal services are provided at the maternity units. Male partners are encouraged to attend antenatal and postnatal sessions with the pregnant women.

At the community level there is a HC II within each parish which serves a number of villages. This is a general outpatient unit that has no maternity unit and, at times, no midwife. At the lowest level (which is HC I) within each village, volunteers from within the community are nominated by members of the community to form Village Health Teams (VHTs). These VHTs are entrusted with taking care of health matters of the village where they live, and they mobilize people for health programs as well as identify and refer individuals who need care. There is no built structure at this level and there are no qualified health staff.

The Kamuli district has only one psychiatric clinical officer (equivalent of a nurse practitioner or nurse prescriber) and a handful of psychiatric nurses. These are all based at the only public hospital and largely work in non-mental health clinics, leaving most of the district with no access to psychiatry personnel. Perinatal women with mental illness are only identified if they are severe enough to be psychotic or suicidal, in which case they are not treated but immediately referred to the regional hospital in the neighboring district of Jinja, sixty kilometers away. Depression and other common mental disorders normally remain undetected and untreated at the primary care level.

### Sample

Study participants consisted of Village Health team members (VHTs), pregnant and postpartum women (within one year of delivery), midwives, general nurses and health managers (Table 1). The district mental health coordinator purposively recruited VHT members from across the district to include those that were most active and likely to have the most knowledge about the health system and community. Twenty VHTs were recruited in two groups of ten. The pregnant and postpartum mothers were recruited from the maternity clinic of the district hospital. Four focus groups were conducted, each with 12 perinatal women. A facilitator presented an overview of the research program and focus group procedures in the hospital’s waiting area, just before the antenatal/postnatal clinic started. Women who were willing to participate were selected by the triage nurse and organized into groups where verbal consent was sought. Eight key informant interviews were conducted with health managers, midwives and general nurses from a wide range of health facilities within the district.

**Table 1:** Demographic characteristics of participants

	Pregnant women focus group	Postpartum women focus group	Village Health Teams	Key informants
	*N* = 24	*N* = 24	*N* = 20	*N* = 8
Age range (years)	18-33	22-42	27-45	25-47
Mean age (years)	22.79	29.83	33.50	33.75
	(SD = 3)	(SD = 2	(SD = 3)	(SD = 2)
Sex				
Male	0	0	10	2
Female	24	24	10	6
Education				
Primary	20	16	0	0
Secondary	4	8	20	0
Tertiary	0	0	0	8
Religion				
Christian	17	19	16	7
Moslem	5	3	4	1
Other	2	2	0	0

### Procedure

Focus groups were conducted in the month of May 2013. Each focus group lasted approximately 90 min and was audio taped. Two facilitators (DK or JN) led the groups, one of whom led the discussion while a second person (an assistant) took notes and recorded the proceedings. The facilitators were graduate specialists in mental health who had experience in conducting focus group discussions and key informant interviews. DK had just completed her PhD thesis using qualitative methods and helped to train the assistants in focus group methodology and note taking. Both facilitators worked at the national referral and teaching psychiatric hospital. The assistants were social worker volunteers with a temporary placement at the Kamuli General Hospital.

### Data collection and tools

An interview guide was used to guide each focus group discussion and key informant interview (Table 2). The interview guide was developed jointly by the authors (JN, DK, JS, FK. SH and CH). Structured, open-ended questions with guided prompts were developed, based on the existing literature and authors’ experience. The core topics for the focus group interviews were (1) barriers and facilitators to accessing mental health care, (2) knowledge and attitudes towards maternal mental disorders, (3) services available for mothers with mental disorders, (4) identified needs for service for maternal mental disorders, (5) feasibility and acceptability of interventions for maternal mental health care and (6) recommendations. The key informant interviews addressed mainly informants’ perception of the problem of maternal mental disorders in the district; causes; existing capacity to detect and manage maternal mental disorders; feasibility of screening; what mental health interventions can feasibly be integrated into maternal health services; availability of supervision for maternal health services; services currently being offered; mechanisms for referral of mothers with mental disorders; existing needs with regard to maternal mental health care; challenges existing or envisaged.

**Table 2:** Focus group and key informant interview guide

• Explore with both pregnant and post-partum women groups:
o Women’s understanding of own mental health needs? Do women perceive themselves to be distressed around pregnancy?
o What kinds of barriers do they face accessing maternal health care?
o What kinds of barriers do they face to accessing mental health care?
o What would be feasible and acceptable forms of intervention for women? For example, group versus individual based interventions?
o Who would be they prefer to deliver intervention? Where? How many sessions? Group-based or individual therapies?
o What other care providers would/could they access?
• With VHTs groups
o What is their understanding about the mental health needs of pregnant and post-partum women?
o What kinds of barriers and opportunities can they identify with regard to these needs?
o What programs or interventions are already in place within the community? Can these be utilized/strengthened/upgraded to include a maternal mental health component?
o Asses the feasibility/accessibility/willingness of this group to provide support to mothers in distress.
• With health worker/health manager key informants:
o Their existing skills and knowledge
o Their training needs
o Their attitudes to task-shifting or integration of maternal mental health care into their existing duties?
o How many visits/sessions are feasible?
o what their own mental health needs/concerns may be

### Data management and analysis

Data were analyzed using the thematic analysis method [25]. Data were transcribed verbatim in the Luganda language and the transcripts were then checked against the audiotapes and scripts. Transcripts were then translated into English and back translated into Luganda by two bilingual authors (JN and DK) to ensure linguistic equivalence. The analyses were performed with the help of qualitative software (Atlas.ti version 6.1) [26]. Both the focus group and key informant data were analyzed together. Two of the authors (JN, EO) independently read through different transcripts each, formulated codes guided by predefined themes in the interview guide as well as by the issues emerging from the data. The two authors met and discussed the codes and emerging themes from the transcripts. A codebook was formulated from the agreed codes and code definitions. One author (EO) then read and coded all of the interview transcripts. After coding, data segments corresponding to major themes were retrieved using Atlas.ti. The retrieved segments of coded data were shared among the authors. Each author read segments allocated to them and wrote memos describing their understanding of what the data said in relation to the study objectives. Finally, data were explored to identify key themes and relationships between themes. This approach was intended to facilitate innovative ways of looking at the data.

### Ethical considerations

The study was approved by the School of Medicine Research and Ethics committee (SOMREC), Makerere University College of Health Sciences, and the Uganda National Council of Science and Technology (UNCST). Permission to conduct the formative work of the Program for Improvement of Mental Health care (PRIME) in Uganda, a multi country research consortium, was obtained from District administration. Verbal group consent was obtained at the beginning of each group discussion and written individual consent before each in-depth interview.

## Results

Four broad themes were identified: i) knowledge and attitudes about maternal mental illness, which describes knowledge about causes and effects of perinatal mental illness and availability of services for women with perinatal mental illness, ii) Patient, household health system and community related barriers to accessing mental health services for women with perinatal mental illness, iii) the perceived feasibility and acceptability of delivering maternal mental health services at primary health care level and iv) proposed recommendations for maternal mental health services.

### Knowledge and attitudes concerning perinatal mental health problems

We explored knowledge about mental health problems among pregnant and postnatal women. The women were aware, to som**e** extent that mental health problems occur during pregnancy and after child birth. Women attending the antenatal clinic described a range of behavioral symptoms of mental health problems in a mother:

*Her ways change (embeeraze zikyuka) …She can do things she doesn’t do normally. She may become very talkative and yet normally she is a quiet person. Another person could be there peacefully with her husband. They never quarrel. But then the minute she gets pregnant they start quarreling and even fight. (***Focus group: pregnant woman)**

The mothers also had an explanation for causes of mental ill health. They believed that ‘thinking too much’ or ‘not loving their husband enough’ could result in one developing mental health problems.

*Sometimes it is due to thinking a lot. She just gets fed up (yekyawa bwekyaye) and doesn’t want to talk to anyone. It could be that she has lost love for the husband* (**Focus group: pregnant woman**)

The mothers also attributed perinatal mental ill health to lack of support and neglect of women by their male partners,

*For me, I think it could be neglect from the husbands***. (Key informant: Nurse)**

*Actually, I think the problem is big, only that we have not really followed up. It seems they have problems at home with their husbands. Some women are not facilitated to come for medical care. Some husbands drink a lot and the women end up having a lot of stress***(Key informant: Clinician)**

Many of the women said they did not really know what the effects of mental illness in the mother or their baby would be, but they believed that it could cause the mother to be “struck by pressure”. This was explained to mean being extremely distressed to the extent of losing consciousness. Suicide was also mentioned as a possible consequence of such illness in a mother.

*She may get ‘pressure’ (yandimukuba), sometimes some women strangle themselves* (**Focus group: pregnant woman)**

On the other hand, the health workers believed that these mothers, once mentally ill, are at risk of being sexually abused, neglecting themselves and even neglecting their babies.

*Sometimes the women end up sleeping on verandas or bushes when they become ill and are unable to look after their babies. They can even get raped.***(Key informant: Midwife)**

It was evident therefore that participants, both the women in the focus groups and key informants, had some ideas that mental ill health occurred among perinatal women. The women in particular believed that the causes of such illness was mainly social and had some explanation regarding the effects of perinatal mental illness on the mother and the baby.

### Barriers to delivery of perinatal mental health services in Kamuli district

Household, health-system and community level factors emerged as important barriers to uptake and delivery of perinatal mental health care. Household barriers revolved mainly around poverty in individual households which led to many women not having enough money to pay for transport to the nearest health facility should they need care. Long distances of up to seven kilometers to the health facilities did not help matters. Distance from health facilities and lack of transport money did not only limit access to mental health services but also to general antenatal care, where mental health problems would be recognized early and managed.

*A mother may be aware of the service, know the advantage of going to a health facility but she doesn’t have transport money. Even if you give a health education talk, she will be aware but she will not have transport.***(Focus group: Village Health Team member*)***

Since most women in Kamuli tend to be unemployed and without any income, the support of their male partners is critical. However, the women reported that their male partners were often unwilling to support them to access health services. Men were said to not only fail to support their partners to attend antenatal care but sometimes to refuse to grant them permission to access such services at all, even on their own. This was highlighted by one pregnant woman in a group.

*There are some men who don’t want to come to hospital with their wives. He tells her that if you don’t go to hospital, can’t you survive, can’t you deliver, will you die? He refuses her to go to hospital* (**Focus group: pregnant woman).**

This reluctance to support perinatal women was believed to arise from the men’s fear that if their partners went for antenatal or postnatal services, the men themselves may be required to accompany them and to take an HIV test. Male partners are particularly encouraged to attend antenatal clinics, where there is routine testing for HIV/AIDS for the couples.

Relatedly, the practice of polygamy, which was reported to be common in Kamuli district, influences the amount of support that a male partner may give to his pregnant partner. One midwife reported:

*A man of 40 years marries another woman and starts a new family. And when the first wife asks for help from the man, the man refuses and so she delivers in the village because they cannot afford anything for this woman.(***Key informant: Midwife)**

In addition to household related barriers, there were health facility-related challenges that were reported to affect the women’s access to maternal mental health care. Low staffing levels at the health facilities, including the district hospital was cited as a key barrier. Existing staff in the health facilities felt they did not have enough time to assess women for mental health problems, as described by one midwife.

*We are only 3 midwives and for one midwife to deal with 80 patients is very hectic. And this issue of male involvement, makes our work to be too much*. So *we do not have enough time to handle mental health issues. (***Key informant: Midwife)**

As a result of the scarcity of trained midwives, and the large numbers of patients that attend the maternity units, the time spent by a mother with a midwife was reported to be very limited. Furthermore, owing to the high levels of poverty and low levels of support from their male partners, mothers seldom return for further care after childbirth, limiting the opportunity to quickly identify those at risk of mental ill health.

*After normal delivery, most mothers ask to be discharged immediately, yet such problems of mental health may manifest after leaving hospital.***(Key informant: Health manager)**

Exacerbating the low levels of staffing, the available midwives reported that they were not adequately trained to handle mental health problems in mothers. Furthermore, their maternity units did not even have the necessary medication to treat mental illness should this be needed. The midwives believed that the general nurses training addressed mental health better than midwifery training and yet the general nurses do not usually provide mental health care either, as one midwife elaborated.

*Midwives are not well equipped with mental health knowledge and skills. If midwives were trained on mental health they could do a better job and with the issue of not having knowledge and drugs, this makes work not easy.***(Key informant: Midwife)**

It was clear, therefore, that there were logistical, health organizational as well as individual health worker competency issues that needed to be addressed to make the provision of perinatal mental health care possible.

In the community it appeared that there were low levels of identification and referral of pregnant and postnatal women in need of mental health services which impeded their access to care. Although there are VHTs within communities who are expected to be a link between the community and the health facility and to mobilize and refer patients for treatment, some health workers thought that these VHTs were being underutilized. As such, there was little or no monitoring, identification or referral of mothers in need of mental health services and yet the midwives lacked the means and the time to reach out to these mothers. One midwife lamented:

*VHTS are always around the health center but they have little work to do because they are not aware of what to do. Over 40 VHTS are attached to our health center!* (**Key informant: Midwife)**

The performance of the VHTs observed by the key informants was thought to be due to little or no support or coordination by the health management in the district. These VHTs are volunteers and therefore are not paid for the work they do. Health workers believed that if VHTs are well coordinated and paid this would motivate them to do more to identify and refer mothers that need care. One general nurse said:

*At times, the VHTs ask for facilitation in terms of money and if they are not given money they give up. At times due to political influence, when they ask for help, they are shut down.****(*Key informant: General Nurse*)***

The existence of the informal community health system was, therefore, seen as an opportunity to be harnessed. However, there was a need to better support and utilize the VHTs to improve the identification and referral of perinatal women in need of mental health care.

Another barrier to access to maternal mental health care at the community level was reported to be negative attitudes and beliefs about the causes of mental illness in a pregnant or postpartum woman. This was believed to hamper health seeking and referral of perinatal women with mental health problems. The community was said to associate mental illness, especially during pregnancy, with witchcraft. This in turn limited the uptake of services as reported by one key informant:

*Cultural influences can be a hindrance. Generally, here, people believe so much in witchcraft. Before someone is brought to hospital they are taken to the witchdoctors. (***Key informant***:***Clinician**)

This belief that mothers who are mentally ill are bewitched causes significant stigma towards the ill mother. The community was reported as viewing these ill women as useless, so that no time should be wasted on them. As such nobody took care of them, as expressed by one clinical officer:

*You may find that people take such people as useless so we need to sensitize …… on this (***Key informant: Clinician)**

### Feasibility and acceptability of delivering perinatal mental health services in primary care settings in Kamuli district

We explored the perceived feasibility and acceptability of incorporating methods of identifying and treating perinatal women with mental health problems at both the community and health facility level. This would ensure that more women that need help are identified and referred for care. These methods included i) screening by health workers at health facilities and ii) VHT community identification and iii) counseling of mothers at the health facility.

Health workers had mixed reactions regarding the feasibility of screening for perinatal mental disorders in their clinics. While some believed that screening would be feasibly done in the same way that it is used to identify patients with tuberculosis and HIV/AIDS in the district, others had reservations. The latter feared that introducing screening for mental health problems would increase their work burden which is already unmanageable. As one general nurse reflected:

*Regarding the use of questionnaires, most mothers do not know how to read. I do not think that these questionnaires will work. There is a heavy work-load. (***Key informant: General Nurse)**

Furthermore, health worker key informants noted that primary health care facilities lacked people with the necessary skills to deal with perinatal mental health problems. As such they believed that treating such women in primary care facilities would not be possible. Women with mental health problems, once identified, are referred to the district or regional hospital where it is believed they can access mental health services, especially medication. One midwife voiced her concern as below:

*We do not admit people with mental health problems, we just refer them. We do not have doctors. I was not trained to treat people with mental health problems. We do not have drugs for people with mental problems. (***Key informant: Midwife)**

The VHTs, on the other hand, were enthusiastic about incorporating identification and referral of perinatal women with mental health problems into their routine work. The VHTs reported that detection and referrals for mental health problems was already part of their role. However, it was acknowledged by some that they are limited by the fact that they are not appropriately trained to provide community mental health care. They suggested that training would make it easier to incorporate perinatal mental health into their work in the community.

*I think that as we continue doing voluntary work we need some knowledge of counseling. If we are taken through that training of counseling, I think that can be integrated in our work***(Focus group: Village Health Team member).**

On the acceptability of treatment, group counseling was believed to be an acceptable modality of treatment for perinatal women, especially in rural areas. The rural women in Kamuli were reported to usually meet in groups and talk about the issues that concern them in daily life. Group counseling would therefore fit naturally into this already existing traditional way of dealing with difficult issues in their life. According to the mothers and VHTs, it would be easy to bring mothers together to share their experiences and reduce their stress.

*That is the best way to do it. Some of the issues they share when they are together can help them to learn from one another; (***Focus group: Village Health Team member)**

*……….and you can go in that group when you are stressed and by the time you leave the group, stress is off (***Focus group: postpartum woman***).*

For the midwives, group counseling would not only be acceptable because it is close to tradition, but also because it is convenient for them as service providers. Group counseling would be efficient in terms of saving midwives time, which is already constrained by huge workloads.

*Group counseling would be better in educating mothers on mental health because you may find that most at them have similar problems. It is also less time consuming.* (**Key Informant: Midwife)**

### Services available for mental health care

A midwife reported that she was not aware of any perinatal mental health services in primary health care facilities within Kamuli district except at the district and regional referral hospitals. The latter is in a neighboring district. As a result mothers are referred away from the lower health facilities to the regional hospital for help once identified to have or to be at risk of mental illness, for fear of the mother’s mental illness becoming too complicated for the midwives to manage. She stated:

*If we have someone that has had any mental problem in the previous pregnancy, we refer them to the main hospital. We do this during health education such that she does not bring us problems.***(Key Informant: Midwife)**

It seemed therefore that there are no available mental health services within the community for perinatal women to access. Traditional healers appear to form the basis of mental health care for the perinatal woman in the community largely because of how the cause of such illness is understood.

### Recommendations from participants

Several recommendations were made by the study participants. These included:

a) Making mental health medicines available in maternity units where they are not currently stocked; b) that there needs to be provision of food for mothers needing admission, otherwise families would opt not to bring these mothers for care. Key informants reported that mothers had difficulty getting food in case they needed to be admitted for severe mental illness due to the severe household poverty. c) The need to bridge the knowledge gaps among midwives through training them in screening, identification and treatment of maternal mental health problems was raised numerous times by a range of respondents; d) Ensuring that resources are provided to enable mental health services to be provided alongside general maternal health care; and e) empowering the VHTs through training in order to increase community sensitization, follow up and support for mothers at the community level.

## Discussion

In this qualitative study from a rural Ugandan district, key barriers and facilitators for integrating maternal mental health care into existing maternal health services were identified. Participants reported inadequate provision of maternal mental health care in Kamuli district. Responses indicated significant gaps in knowledge about these problems amongst both perinatal women and health care providers. Attitudes towards mothers with mental illness were negative, even among health workers. Numerous barriers were believed to limit access to mental health care for mothers and these included: poverty, lack of transport means to health facilities, poorly trained health workers, poor partner support and stigma and discriminating attitudes towards affected mothers. The primary health care facilities were not thought to be adequately equipped to respond to maternal mental health problems. Both primary health workers, as well as perinatal women, acknowledged the need for interventions to respond to this neglected public health problem and identified the barriers that need to be overcome.

### Barriers to delivery of maternal mental health services

Maternal mental health services in Kamuli district are perceived to be inadequate. This was reported to be due to a range of barriers related to the household, health system and community as well as knowledge about mental health. Babalola and Fitusi have reported similar findings [27].

#### Knowledge related barriers

In this study it was strongly evident that there was low levels of perinatal mental health literacy and this was reported by the women and reiterated by the health worker participants. The term “Mental Health Literacy” was defined by Jorm and colleagues as “Knowledge and beliefs about mental disorders which aid their recognition, management or prevention” [28]. Jorm and colleagues further elaborated the other elements of mental health literacy which include knowing how to seek mental health information, knowledge of risk factors and causes of mental ill health as well as knowledge of professional help available for the treatment of mental illness. Other elements include having the attitudes that promote recognition and appropriate help-seeking. These elements of literacy were evidently inadequate among participants in this study. Knowledge regarding the causes and treatment of depression and psychosis was limited among both perinatal women and health worker key informants. Causation of mental illness in a pregnant or postnatal woman was attributed to social difficulties, especially poverty and having problems with one’s spouse. Help-seeking preferences were mainly to informal providers rather than in the formal primary healthcare system. Similar findings were found in Ethiopia, a country geographically and contextually close to Uganda [29, 30]. Previous studies have reported the negative effect of low mental health literacy on service demand, delivery and utilization [31]. The implications for design of perinatal mental health services, therefore, is that such mental health literacy among pregnant and postpartum mothers should be addressed in order to influence attitudes and help seeking behavior of these women. Working with VHTs in the community may be a good way to improve mental health literacy.

#### Household barriers

Among the household-related barriers were household poverty and poor male partner support. These barriers perpetuate the low utilization of maternal health services in general, which in Uganda ranges between 60 % for one antenatal visit to 30-40 % for any postnatal visit [32]. The two way relationship between poverty and mental illness has been highlighted in previous studies [33]. Poverty limits the ability of individuals and families to access care and this was reported by the pregnant and postpartum women in Kamuli to be the main reason for their inability to get transport to the health centers. Health policies and perinatal service planners in low income settings need, therefore, to bear this reality in mind and ensure delivery of mental health services at the lowest level of care within the community in order to improve access to perinatal mental health care. Similarly, lack of support for a pregnant or postnatal woman by their partner or the father of their child is not only associated with increased prevalence of maternal ill health but also with increased inability to access treatment, as was found in Uganda [34]. The implication of this is the need for involvement of the women’s male partners especially in mental health literacy programs to highlight their role and harness their support.

#### Health system related barriers

There were barriers related to the health system in Kamuli which were both structural and service related. The main structural barrier highlighted in this study was the long distance to health facilities. Owning a car in this rural community is unusual for most families, meaning that pregnant and postpartum women typically have to walk for five or more kilometers or hire a vehicle to reach the health center. However given the reported high levels of poverty, the prohibitive cost of hiring transport may lead women to not attend the health center at all. On the other hand, the service related barriers reported included inadequate staffing of health facilities, burdensome workload for the available health workers, especially midwives, health workers who are inadequately trained in mental health and health workers stigmatizing women with mental health problems. It appears therefore that the health system is not geared to the needs of perinatal women who develop mental illness in Kamuli district. These challenges have been highlighted in other low and middle income countries [35]. In order to increase access to perinatal mental health care therefore the primary health care setting in Kamuli needs to be made responsive to the needs of the users of such care. Previous studies have recommended shifting health care tasks to trained lay health workers as a feasible way of making the health system more responsive in resource limited settings [36, 37].

#### Community attitudes

Barriers reported to exist at the community level were negative attitudes and stigma towards mothers with mental illness as well as the widely held traditional belief that these mothers are bewitched. Stigmatizing attitudes were noted to be held by both the community members and health workers. This stigma causes discrimination towards the mother because it is believed the mother has become useless. Studies related to the effect of stigma and help-seeking have indicated that women may fear judgment, poor treatment and having their baby removed from them. As a result, they may not disclose even the most severe symptoms of mental ill-health [38] making it difficult for them to access care.

Belief in witchcraft was reported to be widespread in the Kamuli community. Such beliefs have been shown to be important in influencing where people go for help [39]. It is not surprising, therefore, that many families will take their mentally ill perinatal family member to a witchdoctor, as indicated by the participants in this study. It was interesting that perinatal mental illness was not attributed to the woman’s own promiscuous behavior during pregnancy in this study, as has been reported in a similar culture in Uganda [40]. The fact that beliefs of this nature are strongly held in the community underscores the need for education of communities about mental health problems especially in perinatal women. This will have the secondary benefit of improving help seeking among the women

#### Feasibility and acceptability of care for mothers in primary care

In this study we assessed health workers’ thoughts regarding the feasibility of using screening questionnaires as part of the routine work to identify women with mental health problems. The impact of using screening scales for mental disorders in routine clinical settings has been found to be limited unless implemented alongside wider system level change [41]. However, it may be beneficial in raising the awareness of pregnant and postpartum women to their own mental health problems. This was found in a study in Maryland, USA, where such a benefit was observed and this increased the likelihood of utilization of available health care services [42]. The challenge in this study was the mixed feelings among key health workers regarding the feasibility of screening. While some believed it would be feasible, others had reservations and cited heavy workloads. This wariness by primary health care workers to do more to identify mothers is not unique to the health workers in Kamuli but was found also in Ethiopia [29]. Nonetheless, this strategy might be worthwhile to consider for enhancing identification and uptake of mental healthcare for mothers. It is important to consider brief screening tools to minimize the burden on health workers.

Group counseling was considered by health workers to be feasible as a modality of treatment for mothers with mental health problems as it was thought to fit in well with the existing culture of women sitting together in the village and discussing the issues that affect them.

#### Perceived needs and recommendations

The need for training of primary health care (PHC) workers and VHTs was a recurring theme across the primary care respondents. This highlights the need to address knowledge and skills gaps among primary care providers in order to improve screening, identification and management of maternal mental health problems. Respondents raised the need to consider and address challenges related to the indirect costs to the woman in treatment, such as cost of transport to the health facility and food while admitted as an inpatient. This points to the role of poverty in limiting access to mental health care for mothers, which cannot be ignored in the design of mental health interventions in rural, low-income settings.

### Limitations

A potential limitation in this study was fear of possible repercussions, for the mothers, of disclosing mental health or social difficulties or any dissatisfaction with the health service. In addition, VHT members may have been reluctant to disclose dissatisfaction with the system for fear of reprisals from the authorities. This could have hindered the free flow of information. These concerns may have existed despite the careful informed consent procedure which detailed the processes for anonymity. We could also have lost some of the meaning of respondents’ experiences and perspectives in the process of translating the transcripts from the local language into English.

## Conclusion

This study highlights the need for maternal mental health care in the rural, low income community in Kamuli district in Uganda. It also underscores some of the key barriers to delivery of maternal mental health services in primary care. These include negative community attitudes and stigma towards mental ill health in mothers; inadequate infrastructure that limits geographical access to treatment services, gaps in the health system, a low level of mental health literacy among both mothers and health workers, inadequate support from a woman’s partner and rampant poverty in families. Many of these factors are similar to those reported in other low income countries. The perceived feasibility of group counseling presents an opportunity for an efficient modality of treatment for mothers who might benefit from it. The feasibility of providing mental health care to perinatal women in primary care in Kamuli therefore would depend on having these barriers addressed and on harnessing the available resources in the district to facilitate the development of accessible and responsive maternal mental health services.

Further research is needed to address the “how” of integrating mental health care within the general maternal health services and how the barriers can be overcome and facilitators harnessed to make such services more feasible and acceptable to the target population. Research is also needed on what outcomes result from such care. The results of this study will inform the development of the perinatal mental health services to be initiated in Kamuli district as part of PRIME. Our findings are relevant to maternal mental health service development not only in Kamuli district but in Uganda as a whole, as well as in other LMIC settings similar to Uganda.
